# Advancing peristalsis deciphering in mouse small intestine by multi-parameter tracking

**DOI:** 10.1038/s42003-023-05631-2

**Published:** 2023-12-07

**Authors:** Anusree Sasidharan, Breman Anil Peethambar, Keerthi Santhosh Kumar, Ashok V. Kumar, Arun Hiregange, Neil Fawkes, James F. Collins, Astrid Grosche, Sadasivan Vidyasagar

**Affiliations:** 1 Department of Radiation Oncology, University of Florida, Gainesville, FL USA; 2 Entrinsic Bioscience, Norwood, MA USA; 3 Department of Mechanical and Aerospace Engineering, University of Florida, Gainesville, FL USA; 4 Food Science and Human Nutrition Department, University of Florida, Gainesville, FL USA

**Keywords:** Gastrointestinal models, Gastrointestinal models

## Abstract

Assessing gastrointestinal motility lacks simultaneous evaluation of intraluminal pressure (ILP), circular muscle (CM) and longitudinal muscle (LM) contraction, and lumen emptying. In this study, a sophisticated machine was developed that synchronized real-time recordings to quantify the intricate interplay between CM and LM contractions, and their timings for volume changes using high-resolution cameras with machine learning capability, the ILP using pressure transducers and droplet discharge (DD) using droplet counters. Results revealed four distinct phases, B_*Phase*_, N_*Phase*_, D_*Phase*_, and A_*Phase*_, distinguished by pressure wave amplitudes. Fluid filling impacted LM strength and contraction frequency initially, followed by CM contraction affecting ILP, volume, and the extent of anterograde, retrograde, and segmental contractions during these phases that result in short or long duration DD. This comprehensive analysis sheds light on peristalsis mechanisms, understand their sequence and how one parameter influenced the other, offering insights for managing peristalsis by regulating smooth muscle contractions.

## Abstract (web summary)

A multi-parameter real time tracking setup that synchronizes intestinal circular and longitudinal muscle contractions, intraluminal pressure, and volume recordings to decipher the parameters influencing peristalsis and luminal discharge in mice.

## Introduction

The complex and intricate motility patterns of the gastrointestinal (GI) tract facilitate the proximal to distal movement of ingested food^1,2^. Peristalsis and segmentation are two distinct forms of motility that facilitate in thorough mixing, digesting, absorbing, and final excreting of foodstuffs^3–5^. Peristalsis involves involuntary contractions of both circular muscle (CM) and longitudinal muscle (LM) layers, propelling chyme from oral to aboral direction. In contrast, segmental contractions driven by CM facilitate food mixing^3,5,6^. The coordinated actions between CM and LM is key for proper food propulsion, and any disruption can lead to GI motility disorders^7^.

GI motility disorders refer to conditions that affect the normal movement of food or digesta through the digestive tract and can affect any part of the GI tract from the esophagus to the anus. The underlying cause may often be considered as unknown or multifactorial, involving a combination of genetic, environmental and lifestyle factors. Although the underlying mechanisms and complex interplay of factors involved in GI motility have been explained in previous studies^7–10^, the management of GI motility disorders is still complicated by an incomplete understanding of the precise mechanisms coordinating GI smooth muscle activity, which are necessary for proper bowel emptying.

Our collective understanding of mechanisms underlying gut motility has improved exponentially from new, innovative ex vivo and in vivo approaches to study GI motility, including: (1) high resolution manometry, which records pressure changes at various locations in the intestine; (2) spatiotemporal mapping, which allows analysis of CM and LM contractions; (3) fluoroscopy and MRI, which provide real-time imaging of gut contractions; and (4) in vivo quantification of electrolyte and fluid flux using a number of experimental techniques^9–14^. Due to technical limitations, however, all these parameters have not heretofore been studied simultaneously. Doing so would be a major scientific advance since it would allow comprehensive assessment of how CM and LM work together to appropriately modulate intraluminal pressure changes, anterograde, segmental and retrograde contractions to result in droplet discharge (DD).

The research described in this communication was designed to conceptualize and develop a sophisticated machine that integrates multiple components and subsystems to allow comprehensive analysis of gut motility. One major goal was to be able to accurately quantify the intricate interplay between CM and LM movements, as well as the precise timing of their contractions for resultant DD. In achieving this goal, we hoped to improve the understanding of how numerous physiological factors such as intraluminal pressure (ILP), peristalsis, segmental and longitudinal contractions, absorptive or secretory state of the intestine, and DD at the aboral end work in tandem to maintain GI motility. This knowledge could ultimately lead to the development of novel therapeutic approaches for GI motility disorders.

## Results

### Droplet discharge primarily occurred in *D*_phase_ or *A*_phase_

In the “Short-D group” [S_D_; short duration droplet discharge occurring in the “During Phase” (phase during high amplitude pressure waves; *D*_Phase_)], the DDs occurred during the *D*_Phase_ lasting 0.16 to 0.63 min (range) with an experimental DD duration divided by standard DD mean (D/Ds) of 0.85 ± 0.05. The “Long-A group” [L_A_; long duration droplet discharge occurring in “After Phase” (phase after high amplitude pressure waves; *A*_Phase_)] exhibited significantly longer duration (range: 0.64–0.98 min), with a D/Ds of 1.09 ± 0.05 (range: 0.98–1.8). L_A_ group exhibited longer durations compared to S_D_ group. Similarly, the “Long-D group” (L_D_; long duration droplet discharge occurring in *D*_Phase_) group had all phases longer than *S*_D_ (range: 0.68–0.81 min) and a significant D/Ds increase (1.24 ± 0.04, *p* < 0.001). L_D_’s *D*_Phase_ was longer than L_A_ (0.27 ± 0.02 min vs. 0.33 ± 0.05 min; *p* = NS). These three discharge groups represent distinct lumen filling stages.

### Pressure contraction strength changes during four phases in S_D_, L_A_ and L_D_ groups

Comparing phases across discharge groups, L_A_ showed a higher pressure contraction strength (*Ps*; amplitude of pressure contraction) in the “Before Phase” (*B*_Phase_; phase before the high amplitude pressure waves) compared to S_D_ and L_D_ with significant difference in S_D_ (0.12 ± 0.01 cmH_2_O vs. 0.22 ± 0.02 cmH_2_O, *p* < 0.001; Table 1). *Ps* change between phases was smaller in L_A_ compared to S_D_ and L_D_. Results suggested substantial *Ps* increase coincided with both *D*_Phase_ and *A*_Phase_ fluid discharges. L_A_ group’s fluid discharge featured a relatively modest *Ps* increase. Nonetheless, whether *Ps* of *D*_Phase_ alone accounted for DD duration remained unclear.

**Table 1:** Gross movement and amplitude strength analysis of ILP (intraluminal pressure; referring to *Pg* and *Ps*), longitudinal muscle movement (referring to *Lg* and *Ls*), edge width 1 diameter (*EW*_1_; referring to *EW*_1_*g* and *EW*_1_*s*), edge width 4 diameter (referring to *EW*_4_*g* and *EW*_4_*s*), and volume (referring to *Vg* and *Vs*) between various phases of the different droplet discharge groups.

		Gross movement					Amplitude strength			
		*B* to *N*_Phase_	*N* to *D*_Phase_	*D* to *A*_Phase_			*B*_Phase_	*N*_Phase_	*D*_Phase_	*A*_Phase_
*Pg* (cmH_2_0)	S_D_	0.05 ± 0.01^a^	0.27 ± 0.06^b^	−0.28 ± 0.06^c^	*Ps* (cmH_2_0)	S_D_	0.12 ± 0.01^a^	0.28 ± 0.04^a^	0.72 ± 0.15^b^	0.19 ± 0.04^a,c^
	L_A_	−0.01 ± 0.02*^,a^	0.16 ± 0.05^b^	−0.08 ± 0.06*^,a,c^		L_A_	0.20 ± 0.02*^,a^	0.26 ± 0.03^a^	0.47 ± 0.04^b^	0.25 ± 0.03^a,c^
	L_D_	0.11 ± 0.02*^,#,a^	0.19 ± 0.03^a,b^	−0.27 ± 0.04^#,c^		L_D_	0.16 ± 0.03^a^	0.31 ± 0.04^a^	0.93 ± 0.25^b^	0.27 ± 0.08^a,c^
*Lg* (mm)	S_D_	0.20 ± 0.04^a^	0.14 ± 0.05^a^	−0.33 ± 0.06^b^	*Ls* (mm)	S_D_	0.51 ± 0.07^a^	0.88 ± 0.12^ab^	1.10 ± 0.14^b^	0.60 ± 0.08^a,c^
	L_A_	0.03 ± 0.03*^,a^	0.06 ± 0.02^a^	−0.09 ± 0.02*^,b^		L_A_	0.50 ± 0.1	0.54 ± 0.07*	0.63 ± 0.07*^,^	0.53 ± 0.06
	L_D_	−0.01 ± 0.02*	0.12 ± 0.12	−0.07 ± 0.06*		L_D_	0.82 ± 0.20	0.94 ± 0.18^#,^	1.27 ± 0.25*^,#^	0.99 ± 0.15*^,#^
*EW*_1_*g* (mm)	S_D_	−0.002 ± 0.004^a^	−0.13 ± 0.02^b^	0.13 ± 0.03^c^	*EW*_1_*s* (mm)	S_D_	0.22 ± 0.02^a^	0.26 ± 0.02^a^	0.14 ± 0.01^b^	0.23 ± 0.02^a^
	L_A_	−0.01 ± 0.01	−0.02 ± 0.02*	0.02 ± 0.02*		L_A_	0.19 ± 0.04	0.20 ± 0.04^#^	0.18 ± 0.03	0.19 ± 0.03
	L_D_	−0.02 ± 0.04	0.02 ± 0.04*	0.03 ± 0.05		L_D_	0.29 ± 0.09	0.33 ± 0.06^,^	0.33 ± 0.07*^,#^	0.31 ± 0.09
*EW*_4_*g* (mm)	S_D_	0.02 ± 0.02^a^	−0.06 ± 0.02^b^	0.01 ± 0.02^a^	*EW*_4_*s* (mm)	S_D_	0.16 ± 0.02^a^	0.18 ± 0.03^a^	0.09 ± 0.02^a,b^	0.20 ± 0.03^a^
	L_A_	0.02 ± 0.01	−0.02 ± 0.02	0.02 ± 0.02		L_A_	0.18 ± 0.04	0.19 ± 0.05	0.12 ± 0.04	0.2 ± 0.05
	L_D_	0.02 ± 0.01^a^	−0.07 ± 0.04^b^	0.02 ± 0.01^b^		L_D_	0.23 ± 0.05	0.25 ± 0.06^,^	0.19 ± 0.05	0.23 ± 0.05
*Vg* (mm^3^)	S_D_	−0.03 ± 0.05^a^	−0.65 ± 0.13^a^	0.59 ± 0.15^b^	*Vs* (mm^3^)	S_D_	9.28 ± 1.57	12.59 ± 2.49	8.56 ± 1.52	9.5 ± 1.91
	L_A_	−0.01 ± 0.08^a^	0.07 ± 0.08*^,a^	−0.1 ± 0.09*^,b^		L_A_	6.41 ± 1.26	6.94 ± 1.48*	7.39 ± 1.68	7.51 ± 1.56
	L_D_	0.27 ± 0.17	−0.12 ± 0.17*	0.13 ± 0.08*^,#^		L_D_	11.74 ± 3.7	17.47 ± 5.49^#^	15.42 ± 4.64	14.73 ± 5.49

Significant differences between S_D_, L_A_ and L_D_ in corresponding phases were calculated using one-way ANOVA followed by Bonferroni for individual comparison: * represents significant differences to S_D_, ^#^ represents significant differences to L_A_; significant differences between *B*_Phase_, *N*_Phase_, *D*_Phase_ and *A*_Phase_ in corresponding variables: different letters (a, b, c) indicate significant differences between phases (*p* < 0.05). The number of data for each comparison are accessible in Supplementary Data^51^.

### Gross pressure contraction changes during four phases in S_D_, L_A_ and L_D_ groups

Gross pressure contraction’s (*Pg*; mean pressure changes between two adjacent phases) influence on DD was examined by analyzing its changes between phases (Table 1). In S_D_ group, *Pg* significantly increased from *B*_Phase_ to “Near Phase” (*N*_Phase_; phase near high amplitude pressure waves, after *B*_Phase_), and from *N*_Phase_ to *D*_Phase_ followed by a decrease from *D*_Phase_ to *A*_Phase_. Maximum increase occurred from *N*_Phase_ to *D*_Phase_ (0.05 ± 0.01 cmH_2_O vs. 0.27 ± 0.06 cmH_2_O; *p* < 0.02, *n* = 18). The decrease in *Pg* from *D*_Phase_ to *A*_Phase_ was significant compared to *B*_Phase_ to *N*_Phase_ and *N*_Phase_ to *D*_Phase_ (−0.28 ± 0.06 cmH_2_O; *p* < 0.001, *n* = 18). This study revealed an association between DD in *D*_Phase_ and high *Pg* from *N*_Phase_ to *D*_Phase_.

Comparing *B*_Phase_ to *N*_Phase_ and *D*_Phase_ to *A*_Phase_, S_D_ (*p* < 0.04) and L_D_ (*p* < 0.002) groups had significantly higher *Pg* than L_A_. *Pg* significantly decreased in all three DD groups in *D*_Phase_ to *A*_Phase_. Despite significant pressure changes between phases, L_A_’s *Pg* remained small, potentially contributing to longer durations between discharges. However, this observation did not fully explain L_D_ discharges at relatively higher *Pg* than L_A_ group. Consequently, *Pg* increase could only account for *D*_Phase_ discharges and not discharge duration.

### Gross longitudinal movement changes during four phases in S_D_, L_A_ and L_D_ groups

The magnitude by which LM moved from the oral end during *B*_Phase_ to *D*_Phase_ (0.20 + 0.14 mm) was ~ equal to the distance moved from *D*_Phase_ to *A*_Phase_ (−0.33 ± 0.06 mm) toward the aboral end.

Although gross longitudinal movement changes (*Lg*; mean longitudinal movement between two adjacent phases) increased from *N*_Phase_ to *D*_Phase_ and rapidly decreased from *D*_Phase_ to *A*_Phase_, the L_D_ showed no significant differences between *B*_Phase_ to *N*_Phase_, *N*_Phase_ to *D*_Phase_ and *D*_Phase_ to *A*_Phase_ (Table 1).

In L_A_ group, *Lg* was lower in all three phases, with significant reduction in *B*_Phase_ to *N*_Phase_ and *D*_Phase_ to *A*_Phase_ compared to S_D_ group, suggesting reduced LM contraction. Similarly, L_D_ group exhibited lower *Lg* in *B*_Phase_ to *N*_Phase_ and *D*_Phase_ to *A*_Phase_ compared to S_D_ group (Table 2). However, L_D_ group showed no significant difference between phases and notably less aboral movement compared to S_D_ group (−0.07 ± 0.06 mm vs. −0.33 ± 0.06 mm; *p* < 0.02). L_D_ group also showed minor aboral movement in *B*_Phase_ to *N*_Phase_ compared to different phases (*p* = NS). Reduced LM movement toward both oral and /or aboral ends in L_A_ and L_D_ groups might contribute to the longer duration of these DDs.

**Table 2:** Frequency comparison of intraluminal pressure (ILP), longitudinal muscle (LM) movement, edge width 1 diameter (*EW*_1_), edge width 4 diameter (*EW*_4_) and volume within the various phases of the different droplet discharge groups.

Frequency (Hz)		*B*_Phase_	*N*_Phase_	*D*_Phase_	*A*_Phase_
ILP	S_D_	0.74 ± 0.01	0.73 ± 0.01	0.72 ± 0.01	0.76 ± 0.03
	L_A_	0.69 ± 0.01*	0.66 ± 0.02*	0.66 ± 0.01*	0.67 ± 0.01*
	L_D_	0.73 ± 0.02	0.71 ± 0.02^#^	0.70 ± 0.01^#^	0.70 ± 0.02
LM	S_D_	0.74 ± 0.01	0.73 ± 0.01	0.72 ± 0.01	0.72 ± 0.01
	L_A_	0.69 ± 0.01*	0.65 ± 0.01*	0.63 ± 0.02*^,#^	0.62 ± 0.02*^,#^
	L_D_	0.74 ± 0.01^#^	0.72 ± 0.02^#^	0.72 ± 0.01^#^	0.71 ± 0.01^#^
*EW*_1_	S_D_	0.74 ± 0.01	0.73 ± 0.01	0.73 ± 0.01	0.73 ± 0.01
	L_A_	0.69 ± 0.01*	0.69 ± 0.01*	0.67 ± 0.01*	0.67 ± 0.01*
	L_D_	0.73 ± 0.01	0.73 ± 0.02	0.73 ± 0.02^#^	0.72 ± 0.01^#^
*EW*_4_	S_D_	0.70 ± 0.02	0.72 ± 0.02	0.70 ± 0.01	0.72 ± 0.01
	L_A_	0.68 ± 0.02	0.65 ± 0.02*	0.65 ± 0.01*	0.67 ± 0.01*
	L_D_	0.80 ± 0.11	0.68 ± 0.02	0.70 ± 0.01^#^	0.71 ± 0.02
Volume	S_D_	0.74 ± 0.01	0.75 ± 0.01	0.73 ± 0.01	0.73 ± 0.01
	L_A_	0.68 ± 0.01*	0.67 ± 0.01*	0.65 ± 0.01*	0.65 ± 0.01*
	L_D_	0.72 ± 0.02	0.72 ± 0.02	0.72 ± 0.01^#^	0.70 ± 0.01^#^

Significant differences between S_D_, L_A_ and L_D_ in corresponding phases were calculated using one-way ANOVA followed by Bonferroni for individual comparison: * represents significant differences to S_D_, ^#^ represents significant differences to L_A_ (*p* < 0.05). The number of data for each comparison are accessible in Supplementary Data^51^.

### Longitudinal contraction strength changes during four phases in S_D_, L_A_ and L_D_ groups

In the S_D_ group, the longitudinal contraction strength (*Ls*; amplitude of longitudinal contractions) progressively increased from *B*_Phase_ to *D*_Phase_, then declined in *A*_Phase_. In S_D_ group, *Ls* in *D*_Phase_ was significantly higher compared to both *B*_Phase_ and *A*_Phase_. Conversely, L_A_ group showed no significant differences in Ls between phases. In L_A_ group, *Ls* in *D*_Phase_ was significantly lower compared to S_D_ group. L_A_ group displayed small non-significant differences in *Ls* between phases. Conversely, in L_D_ group, *Ls* in *D*_Phase_ was significantly higher compared to S_D_ group. Although L_D_ generally had higher *Ls*, it lacked significant phase distinctions, potentially contributing to increased DD duration. Furthermore in L_D_ group, *Ls* was significantly higher compared to L_A_ group in all phases except *B*_Phase_ (Table 1).

### Gross volume and volume strength changes during four phases in S_D_, L_A_ and L_D_ groups

In the S_D_ and L_A_ groups, intestinal gross volume (*Vg*; mean volume changes between two adjacent phases) decreased marginally from *B*_Phase_ to *N*_Phase_ and *N*_Phase_ to *D*_Phase_. S_D_ group showed a significant volume drop from *N*_Phase_ to *D*_Phase_, unlike L_A_ group. S_D_ group showed significantly increased volume in *D*_Phase_ to *A*_Phase_ (Table 1). Conversely, L_A_ group displayed minimal changes, with an increase from *N*_Phase_ to *D*_Phase_ followed by a slight decrease in *D*_Phase_ to *A*_Phase_. This suggests delayed fluid filling and emptying in L_A_ compared to S_D_ group. L_D_ exhibited non-significant volume increments between phases unlike S_D_ group. This explains the delayed lumen filling and prolonged duration of DD in L_D_ group. Taking all phases together, S_D_ (9.99 ± 0.96 mm^3^; *p* < 0.001, *n* = 72) and L_D_ (14.84 ± 2.35 mm^3^; *p* < 0.006, *n* = 32) exhibited higher volume strength (*Vs*; amplitude of volume contraction) compared to L_A_ (7.06 ± 0.73 mm^3^, *n* = 76). These studies showed that irrespective of contraction phase, L_A_ consistently displayed lower *Vs*. Phase-related *Vs* changes for S_D_, L_A_ and L_D_ showed no significant difference (Table 1).

### Gross movement of edge width tracker 1 and 4 changes during four phases in S_D_, L_A_ and L_D_ groups

Gross movement of edge width tracker 1 (*EW*_1_*g*; mean diameter changes between adjacent phases in edge width tracker 1; *EW*_1_) exhibited marginal decrease in *B*_Phase_ to *N*_Phase_ followed by a significant decrease in *N*_Phase_ and *D*_Phase_ in S_D_, signifying proximal CM contraction with DD. Conversely, L_A_ and L_D_ had minimal *EW*_1_*g* changes, suggesting DD without significant CM contractions, explaining delayed emptying.

Gross movement of edge width tracker 4 (*EW*_4_*g*; mean diameter changes between adjacent phases in edge width tracker 4; *EW*_4_) increased slightly from *B*_Phase_ to *N*_Phase_ and, decreased significantly from *N*_Phase_ to *D*_Phase_ in S_D_, implying open lumen becoming closed. A small non-significant increase followed from *D*_Phase_ to *A*_Phase_. L_A_ showed smaller insignificant diameter changes. L_D_ displayed a small but significant diameter decrease from *B*_Phase_ to *N*_Phase_ compared to *N*_Phase_ to *D*_Phase_, suggesting closed to open lumen transition. The decreased *B*_Phase_ to *N*_Phase_ diameter could explain L_D_ duration.

Comparing *EW*_1_*g* and *EW*_4_*g* contractions, a significant diameter decreases in *EW*_1_*g* during *N*_Phase_ to *D*_Phase_ suggested proximal contraction with distal CM relaxation. In S_D_, lumen filling primarily occurred in *D*_Phase_ to *A*_Phase_, where *EW*_1_*g* diameter increased compared to *EW*_4_*g* (Table 2). Similar changes were absent in the L_A_ and L_D_ groups, although *EW*_1_*g* showed greater diameter decrease than *EW*_4_*g*.

### Changes in proximal and distal edge width contraction strength during four phases in S_D_, L_A_ and L_D_ groups

In S_D_, proximal edge width contraction strength (*EW*_1_*s*; amplitude of *EW*_1_ contraction) significantly decreased in *D*_Phase_ compared to *B*_Phase_ (Table 1). This suggests maximum CM contractions during *N*_Phase_ to *D*_Phase_ transition when *EW*_1_*s* was lowest. L_A_ and L_D_ showed stable *EW*_1_*s* with no significant phase differences. L_D_ generally had higher *EW*_1_*s* compared to L_A_ with significance in *D*_Phase_. DD in *D*_Phase_ followed *EW*_1_*s* and *EW*_1_*g* decrease, suggesting a role for proximal CM contraction. Unlike *EW*_1_*s*, mean diameter for all phases was lower in *EW*_4_*s* for S_D_ (0.21 ± 0.01 mm vs. 0.16 ± 0.01 mm; *p* < 0.002, *n* = 72) and L_D_ (0.32 ± 0.04 mm vs. 0.23 ± 0.02 mm; *p* < 0.05, *n* = 32). In L_A_, there was no significant difference between *EW*_1_*s* and *EW*_4_*s*. In L_A_ and L_D_, there was no significant phase differences. In S_D_, distal edge width contraction strength (*EW*_4_*s*; amplitude of *EW*_4_) increased from *B*_Phase_ to *N*_Phase_, decreased in *D*_Phase_, and significantly increase in *A*_Phase_ (*p* < 0.03, *n* = 18) with maximum *EW*_1_*s* and *EW*_4_*s* decrease occurring in *D*_Phase_.

### Frequency measurements during different contraction phases and droplet discharge intraluminal pressure wave frequency

Comparing ILP frequency across phases of DD groups showed no significant differences. However, distinct pattern emerged when comparing S_D_, L_A_ and L_D_ groups (Table 2). Frequency decrease was significant between S_D_ and L_A_ in *B*_Phase_ (0.74 ± 0.01 Hz vs. 0.69 ± 0.01 Hz; *p* < 0.02), *N*_Phase_ (0.73 ± 0.01 Hz vs. 0.66 ± 0.02 Hz; *p* < 0.001), *D*_Phase_ (0.72 ± 0.01 Hz vs. 0.66 ± 0.01 Hz; *p* < 0.003) and *A*_Phase_ (0.76 ± 0.03 Hz vs. 0.67 ± 0.01 Hz; *p* < 0.001). This suggests that frequency decreases as contraction decreases, akin to L_A_. Intriguingly, L_D_ exhibited significantly higher frequencies compared to L_A_ in *N*_Phase_ (0.71 ± 0.02 Hz vs. 0.66 ± 0.02 Hz; *p* < 0.05) and *D*_Phase_ (0.70 ± 0.01 Hz vs. 0.66 ± 0.01 Hz; *p* < 0.04), and were not significantly different from S_D_ group.

### Longitudinal tracker frequency analysis

**Fig. 1:**
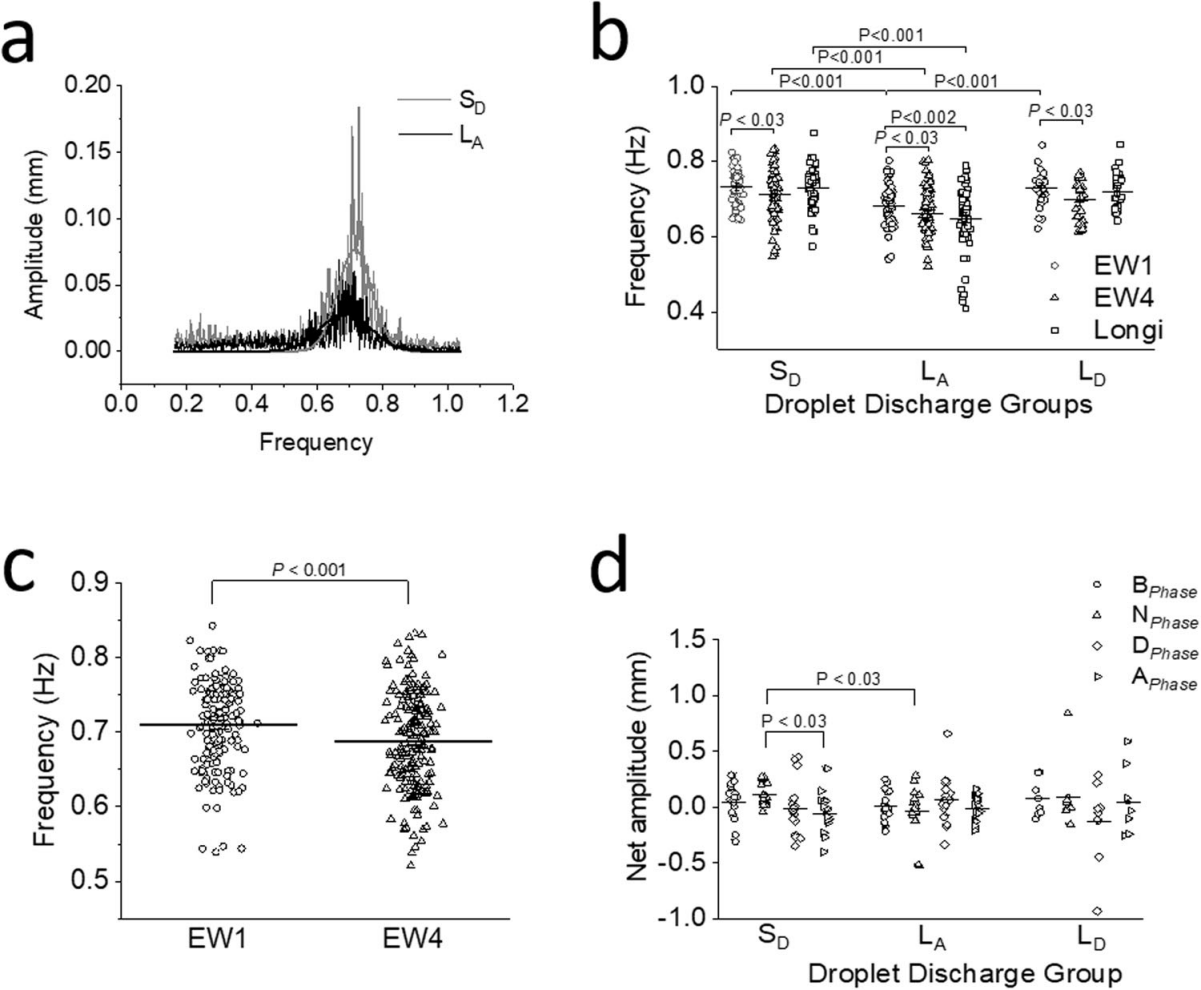
Pattern of frequency, contraction types and net amplitude within droplet charge groups. **a** Representative graph showing fast Fourier transform (FFT) of longitudinal trace. The sinusoidal oscillations of longitudinal contractions for a period were divided into distinct frequency components with their respective amplitude. A high-pass filter with a cut off frequency of 0.2 Hz and an amplitude threshold of 0.02 mm was used. Non-linear exponential curve fit using second order polynomial exponent (Exp3P2) was used to trace along the FFT. The graph shows a shift in the frequency to the left for L_A_ (black tracings) compared to S_D_ (gray tracings), suggesting a decrease in frequency in L_A_. The decrease in frequency was associated with a simultaneous decrease in the amplitude of the contraction. **b** Comparison of *EW*_1_, *EW*_4_ and longitudinal frequency within various discharge groups. *EW*_1_ and *EW*_4_ showed a significant difference in frequencies (Longi) in S_D_ (*p* < 0.03, *n* = 18), L_A_ (*p* < 0.03, *n* = 19) and L_D_ (*p* < 0.03, *n* = 8) discharge groups. Comparing *EW*_1_, *EW*_4_ and Longi of S_D_ with that of L_A_ showed a significant difference. L_A_ showed significant difference between *EW*_1_ and Longi. In L_D_ discharge groups, there was no statistical significance between frequencies for *EW* and Longi. Significant differences were calculated using Kruskal–Wallis followed by Mann–Whitney test for individual comparison. **c** Overall frequency comparison between proximal (*EW*_1_) and distal edge trackers (*EW*_4_) irrespective of the discharge group. Proximal edge tracker have a higher frequency compared to the distal tracker (*p* < 0.001, *n* = 48). Significant differences were calculated using one-way ANOVA. **d** Comparison of net amplitude between *EW*_1_ and *EW*_4_ for the three discharge groups. Net amplitude was anterograde in *B*_Phase_ and *N*_Phase_ in S_D_, followed by retrograde net movement in *D*_Phase_ and *A*_Phase_ with a significant difference between *N*_Phase_ and *A*_Phase_ (*p* < 0.03, *n* = 18). In L_A_, the net amplitude was generally retrograde, with anterograde movement only in *D*_Phase_ (*n* = 19). In L_D_, similar to S_D_, net amplitude was anterograde in *B*_Phase_ and *N*_Phase_, retrograde movements were seen only in *D*_Phase_ followed by a small anterograde movement in *A*_Phase_ (*n* = 8). In L_A_ and L_D_, there are no significant differences between the phases. Significant differences were calculated using Kruskal–Wallis followed by Mann–Whitney test for individual comparison.

Comparing frequencies of LM contractions within S_D_, L_A_ and L_D_ phases showed no significant differences (Table 2), suggesting consistent pacemaker activity across discharge group phases. However, overall frequency and mean amplitude analysis using fast Fourier transform (FFT) revealed higher values in S_D_ than L_A_ (Fig. 1a, b). Frequencies between S_D_ and L_A_ phases displayed significantly lower values in L_A_ group across all phases.

Similarly, L_A_ exhibited significantly lower frequency in all phases compared to the L_D_ group (Table 2). There was no significant difference in longitudinal frequencies between S_D_ and L_D_ phases. In summary, these findings suggest that when the intestinal lumen is mostly empty (L_A_), the contraction frequencies are lower across all four phases. Conversely, in S_D_ and L_D_ the frequencies are higher across all phases.

### Proximal edge width tracker frequency analysis

Comparing frequencies within S_D_, L_A_ and L_D_ phases of *EW*_1_ showed no significant difference. However, significant distinctions emerged between discharge groups. S_D_ exhibited higher frequency compared to L_A_ across all phases (Table 2). Comparing L_A_ and L_D_, L_A_ displayed significantly lower frequencies in *D*_Phase_ and *A*_Phase_. While *B*_Phase_ and *N*_Phase_ in L_A_ displayed lower frequencies compared to L_D_ but was not significant. These studies showed that lower frequencies in the L_A_ group aligned with minimal diameter changes between phases (Table 2), suggesting CM contraction frequency has a potential role in fluid discharge dynamics.

### Distal edge width tracker frequency analysis

Frequency analyses of S_D_, L_A_ and L_D_ revealed no significant difference between phases (Table 2). Comparing S_D_ and L_A_, L_A_ showed significantly lower frequencies in *N*_Phase_, *D*_Phase_ and *A*_Phase_. Similarly, L_A_ exhibited lower frequencies compared to L_D_ in all phases, with a significant difference in *D*_Phase_. However, there were no significant differences in frequency between S_D_ and L_D_ in all four phases.

When comparing overall *EW*_1_ and *EW*_4_ frequencies, a significantly higher frequency was observed in *EW*_1_ (0.71 ± 0.01 Hz vs. 0.67 ± 0.00 Hz; *p* < 0.001, *n* = 180; Fig. 1c). This frequency difference was consistent across various phases of the contractions for S_D_, L_A_ and L_D_ but decreased significantly in L_A_ which prolonged the time for discharge, particularly in *EW*_1_, highlighting the significance of proximal contractions in fluid propulsion dynamics.

Additionally, the frequency pattern indicated stronger proximal contractions, favouring anterograde propulsion. Reduced *EW*_4_ frequency relative to *EW*_1_ could be influenced by the steady oral fluid flow from the peristaltic pump, contrasting with variable fluid flow in aboral end because of pooling and emptying. This differential exposure to fluid flow could affect contraction frequency and strength.

Comparing LM and *EW*_1_ frequencies, S_D_ and L_D_ showed no significant difference across phases. In L_A_, LM frequency significantly decreased compared to *EW*_1_ in *N*_phase_ (0.65 ± 0.01 Hz vs. 0.69 ± 0.01 Hz; *p* < 0.04, *n* = 19) and *A*_phase_ (0.62 ± 0.02 Hz vs. 0.67 ± 0.01 Hz; *p* < 0.04, *n* = 19).

### Anterograde, retrograde, and segmental contractions

#### S_D_ group

The “net amplitude” increased from *B*_Phase_ to *N*_Phase_ (0.05 ± 0.04 mm vs. 0.12 ± 0.02 mm), and declined during *D*_Phase_ (−0.01 ± 0.05 mm) and *A*_Phase_ (−0.05 ± 0.04 mm) with a significant difference between *N*_Phase_ and *A*_Phase_ (*p* < 0.03) (Fig. 1d). This indicates that strong proximal contractions facilitate DD, with anterograde contractions during *B*_Phase_ to *N*_Phase_, while retrograde contractions appeared in *D*_Phase_ and *A*_Phase_.

#### L_A_ group

“Net amplitude” was relatively lower compared to S_D_. Retrograde contractions were noted in *N*_Phase_ (−0.03 ± 0.05 mm) and *A*_Phase_ (−0.01 ± 0.02 mm). Low amplitude anterograde movements occurred in *B*_Phase_ (0.01 ± 0.03 mm) and *D*_Phase_ (0.07 ± 0.05 mm), without significant differences between the four phases (Fig. 1d).

#### L_D_ group

L_D_ had larger amplitude contractions than L_A_. L_D_ exhibited anterograde contractions in all phases except *D*_Phase_, marked by retrograde contractions (Fig. 1d), without any significant differences between phases.

#### Comparing S_D_, L_A_, and L_D_

L_A_ had lower contraction amplitude with a significant difference only in *N*_Phase_ (0.12 ± 0.02 mm vs. −0.03 ± 0.05 mm; *p* < 0.004, *n* = 18 and *n* = 19, respectively; Fig. 1d) when compared to S_D_. No significant differences within phases of S_D_ and L_D_ groups were observed.

These findings suggest anterograde contractions precede DD. Variable “net amplitude” between phases in L_A_ and L_D_ discharge groups highlights the complexity of intestinal motility. Strong proximal CM and LM contractions and volume decrease in *B*_Phase_ to *N*_Phase_ and *N*_Phase_ to *D*_Phase_ are responsible for shorter DD duration, anterograde contraction in S_D_, while lower amplitude and predominantly retrograde contractions in L_A_ might contribute to longer DD durations.

## Discussion

Functional imbalance in GI motility is closely linked to numerous human ailments such as achalasia, dyspepsia, gastroparesis, constipation, IBS, chronic intestinal pseudo-obstruction^15^. The current understanding of intestinal peristalsis relies on in vitro and in vivo techniques including manometry and spatiotemporal mapping, but each technique has its own limitations^9,10^. Technical limitations have hindered quantitative analysis of smooth muscle layer movements affecting ILP and DD. In this study, a multi-spectrum image capture system, ILP transducers, Vernier Drop counters and associated proprietary software programs enabled real time acquisition of quantitative data on intestinal motility and DD. Specifically, the technology achieved: (1) Quantification of frequency and amplitude for both, high and low amplitude pressure waves; (2) Continuous monitoring of diameter and volume changes for luminal content accumulation and propulsion; (3) Characterizing intestinal contractions as anterograde, retrograde or segmentation; (4) Establishing “net amplitude” to assess overall contraction strength and direction; (5) Relationship to DD using various motility parameters; and (6) Establishing absorptive or secretory state of the intestine but is not part of this study, as intestinal segments were perfused with ringer solution and exhibited net absorption. Future comparisons between formulations will consider net fluid output, impacting both, motility and, absorption or secretion. Overall, this is an approach that simultaneously uses all these parameters to study the mechanisms of gut motility.

Interstitial cells of Cajal (ICCs) are specialized pacemaker cells that initiate and propagate slow electrical waves regulating smooth muscle contraction frequency in response to force^16–18^. Various ICC types are classified by their GI tract location^19^. They can be interconnected multipolar cells (myenteric region) or bipolar without interconnections within CM and LM layers^17,18,20^. ICC communicate directly via gap junctions and chemical signaling^18^. ICC initiate oscillations, initially synchronized proximally, but increasingly desynchronized aborally^17^. ICC form networks such as the Myenteric plexus situated between the CM and LM layers, coordinating peristalsis and food propulsion, or the Meissner’s plexus in the submucosa, coordinating local reflexes in response to various GI stimuli, and modulating peristalsis, vasomotor activity, absorption and secretion^19^.

In this study, CM and LM contractions were referenced to four phases of pressure tracing (*B*_Phase_, *N*_Phase_, *D*_Phase_ and *A*_Phase_) to clarify LM’s role in initiating CM contractions, and the resulting volume and pressure changes leading to DD. In S_D_ group, increased *Lg* occurs through *B*_Phase_*, N*_Phase_ and *D*_Phase_, followed by CM contraction. This could be explained by CM fiber gathering and associated ICC proximity, facilitating signal transmission and contraction. In dog ileum, LM played a crucial role in transmitting electrical signals to CM upon distention^21,22^. Similarly, findings in human esophagus revealed that LM contractions precede CM contractions, and last longer^23,24^. *EW*_1_ began to contract in *B*_Phase_ to *N*_Phase_, while *EW*_4_ continued diameter increase suggesting relative aboral CM relaxation while LM contraction peaked ahead of CM contraction. Maximum increase in ILP followed *EW* contraction and decreased volume in *N*_Phase_ to *D*_Phase_, rather than LM tracker movement, suggesting ILP changes as a reflection of CM contraction. Similar observations in other studies on intestine and esophagus have shown concurrent contraction and relaxation of CM and LM layers^25,26^.

**Fig. 2:**
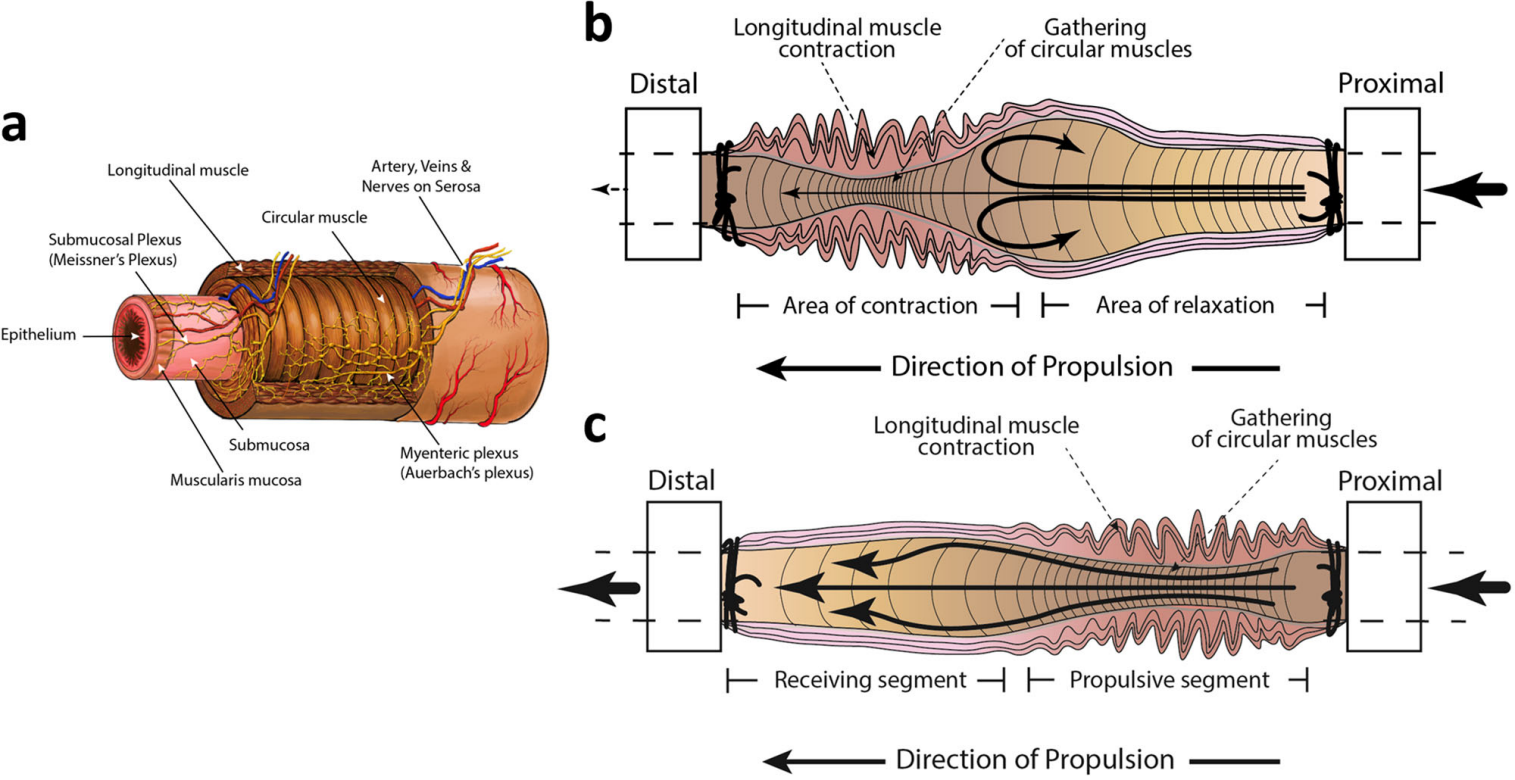
Illustration of the small intestine with organization of circular and longitudinal muscles, and the enteric nervous system, and schematic representation of the intestine showing fluid filling and muscle contractions for droplet discharge. **a** The illustration shows the entry of artery, veins and nerves from the mesentery through the serosa and the distribution of the myenteric plexus between the longitudinal and circular layers of smooth muscle. The orientation of the myenteric plexus is such that the ganglia (collection of nerve cell bodies) are stretched circumferentially and inter-ganglionic fibers run longitudinally, parallel to the longitudinal muscle fibers to allow transmission of signals and coordination of muscle contractions without exerting excessive traction or tension on the nerve fibers. Meissner’s plexus or submucosal plexus is a network of nerve fibers and ganglia located in the loose connective tissue of the submucosal layer of the GI tract. Receptive nerve endings from Meissner’s plexus projects into the mucosa as intrinsic primary afferent neurons and transmit the signals to submucosal neurons or Myenteric plexus. **b** Figure showing longitudinal muscle contraction with gathering of circular muscle rings (muscle fibers) toward the distal end that results in contraction with narrowing of the lumen at the aboral end, favouring fluid-filling. Fluid filling leads to ballooning proximal to the contracted region (bold arrows). Intestinal flow is reduced during the filling stage (thin arrow). Arrows within the lumen depicts more retrograde movement. **c** Figure showing longitudinal muscle contraction (parallel lines) with gathering of circular muscle rings (muscle fibers) and narrowing of the lumen at the proximal end that favors droplet discharge (bold arrows). Arrows within the lumen suggest more anterograde movement. **a** was created using Sketchbook® App in iPad (7th generation) and imported to Adobe Illustrator version 27.1 (2023) for final adjustments and labeling, while (**b**, **c**) were drawn using Adobe Illustrator version 27.1 (2023).

In this study, *EW*_4_ was associated with a more relaxed state compared to *EW*_1_, and this combined with a relatively higher contraction frequency in the proximal regions, favored more anterograde contraction, irrespective of the DD type. Similar observations were made in animal and human showing that GI tract’s distal regions progressively exhibit reduced frequency and contractions^27–31^. Increased frequency and contraction in proximal segments may be necessary for mixing and propelling digested material in the upper GI tract. The frequency of contractions between LM and *EW*_1_ did not significantly differ in S_D_ and L_D_ but showed a significantly lower frequency in L_A_ groups. Similar findings were observed in studies done in human ileal tissue^32^. This explains the decreased frequency, and LM and CM contractions when the lumen is mostly empty, as fewer stretch reflexes are initiated to stimulate the myenteric plexus (Fig. 2a).

The “net amplitude” displayed more anterograde contractions and fluid emptying when distal tracker contractions lagged the proximal tracker contraction. Segmental contractions with no fluid emptying occurred in the absence of a lag between the two trackers. This is evident in S_D_ group, where *B*_Phase_, *N*_Phase_ and *D*_Phase_ were anterograde with a significant difference between *B*_Phase_ and *A*_Phase_. Fluid accumulated toward the aboral end, leading to droplet formation. Although, DD happened in *D*_Phase_ or *A*_Phase_, the fluid accumulation commenced as early as *B*_Phase_ continuing through *N*_Phase_ and the early part of *D*_Phase_. Eventually, the droplet’s weight overcame cohesive forces, causing DD. Thus, anterograde movements occur during fluid emptying phases and shifted to net retrograde movement during fluid filling phases.

Studies suggest that ICCs decide frequency and character of propagative muscle contractions^33^. The proximal edge trackers recorded a diameter reduction from *B*_Phase_ to the end of *D*_Phase_, after which the diameter began to increase progressively and peaking in *D*_Phase_ to *A*_Phase_. *EW*_4_ showed a relatively smaller reduction, indicating less gross contraction. Studies using rat small intestine, showed that proximal CMs were more sensitive to cholinergic drugs and active stress when compared to distal CM contraction^34^.

The decrease in *Pg* and *Ps* during *A*_Phase_ correlated with decreased *Lg*, signifying LM movement toward the distal end, accompanied by increased *Vg* and *Vs*. Following the peak contraction of *EW*_1_, LM contracted aborally in *D*_Phase_ to *A*_Phase_ and *A*_Phase_, associated with increased *EW*_1_, while *EW*_4_ showed only a minor increase. During this phase, the intestinal segment volume increased substantially, indicating fluid filling as LM contracts at the aboral end initiating contraction of *EW*_4_. Subsequently, LM moved toward the oral end as the volume continued to fill, and CM relaxed. This suggests that LM contraction is vital for initiation of contraction at both, oral and aboral ends. However, to sustain the CM contraction at either end, LM contraction may not be necessary (Fig. 2b, c).

In L_D_ group, proximal LM movement was reduced in *N*_Phase_ to *D*_Phase_ compared to S_D_ group but was not significant. *EW*_1_ did not show a significant difference between various phases, but was significantly lower compared to S_D_. These contraction parameters explained the relative retrograde movement observed in *D*_Phase_ of L_D_ group, and the relatively longer time for DD. Both, L_A_ and L_D_ groups had preceding short duration discharges. L_A_ group had a longer DD duration compared to L_D_ group. Consequently, the L_A_ group had less time to fill the lumen before the next discharge, explaining why L_D_ group had a higher intestinal volume, significantly in *D*_Phase_ to *A*_Phase_. In S_D_, intestinal volume increased, likely activating the stretch reflex for strong contractions. In L_A_, delayed filling resulted in less stretch reflex activation, manifesting as lower muscular contractions. L_D_ was intermediate with some intestinal volume increase and potentially stronger stretch reflex and muscle contractions than L_A_, but less than S_D_.

Current management of GI motility disorders, including IBS aims to alleviate symptoms rather than addressing the root cause^35,36^. Most constipation drugs work by modifying fluid balance i.e., by decreasing absorption and increasing secretion, thereby increasing the fluid volume in the gut lumen^37,38^, but they do not address the primary alterations in GI motility. The limited effectiveness in altering the disease’s natural course can be attributed to technical limitations in understanding the complex and simultaneous interactions between ILP, CM and LM contractions and intestinal evacuation. The multi-parameter tracking setup developed in this study overcomes these limitations and gives a quantitative analysis of the complex muscular events governing GI motility and thereby facilitating the development and evaluation of drugs targeting intestine’s natural smooth muscle rhythm to achieve proper bowel evacuation.

## Methods

### Experimental mouse model

**Fig. 3:**
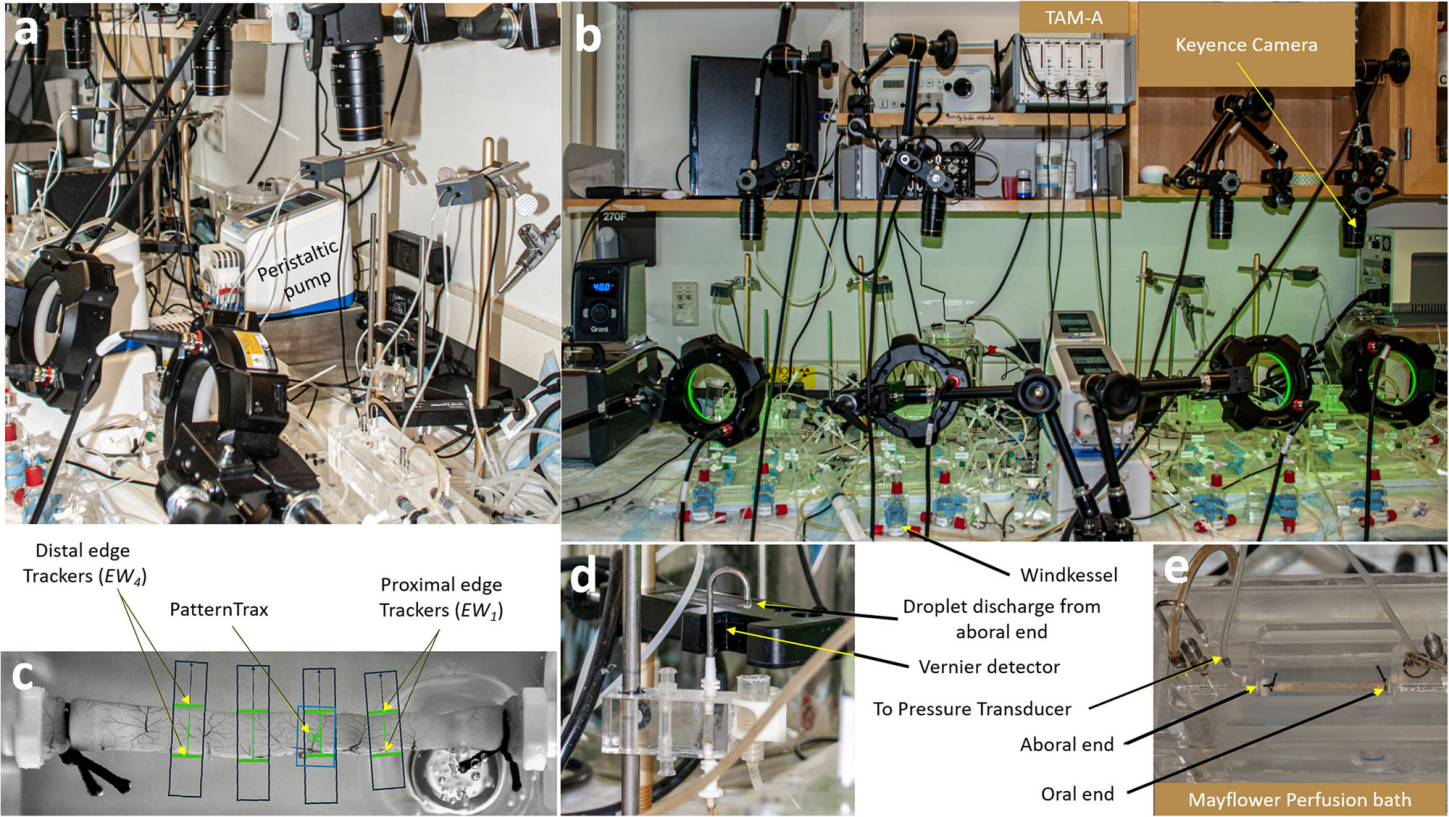
The experimental setup for studying peristalsis: **a** Lateral view, **b** Front view. PLUGSYS Transducer Amplifier Modules-A (TAM-A, Harvard apparatus, USA), CVX400 series vision system with multi-spectrum LumiTrax light (Keyence, USA) and infrared-LED sensor drop counters with LabQuest Mini interface (Vernier instruments, USA) are used to measure intraluminal pressure, diameter changes, longitudinal movements, and fluid output in intestinal segments. **c** Representative image showing jejunal segment mounted in tissue perfusion bath. Jejunal segment with 4 digital edge width (*EW*) trackers and one PatternTrax placed along the length of the intestine. *EW* trackers track the diameter change with time. PatternTrax measures the longitudinal movement with time using surface characteristics on the intestine such as blood vessels. **d** The luminal perfusate passing through the intestinal segments were made to fall through a specified area of the drop-counter so that the LED light falling on the detector is blocked, resulting in the generation of a digital signal that is then captured by a data collection interface. The data are converted into volume and expressed in ml/min by the data acquisition program. **e** Intestinal segments were perfused in water-jacketed tissue bath (Mayflower, USA). The oral and aboral end of the tissues are connected to the pressure transducers and the pressure recordings obtained were amplified using TAM-A.

**Fig. 4:**
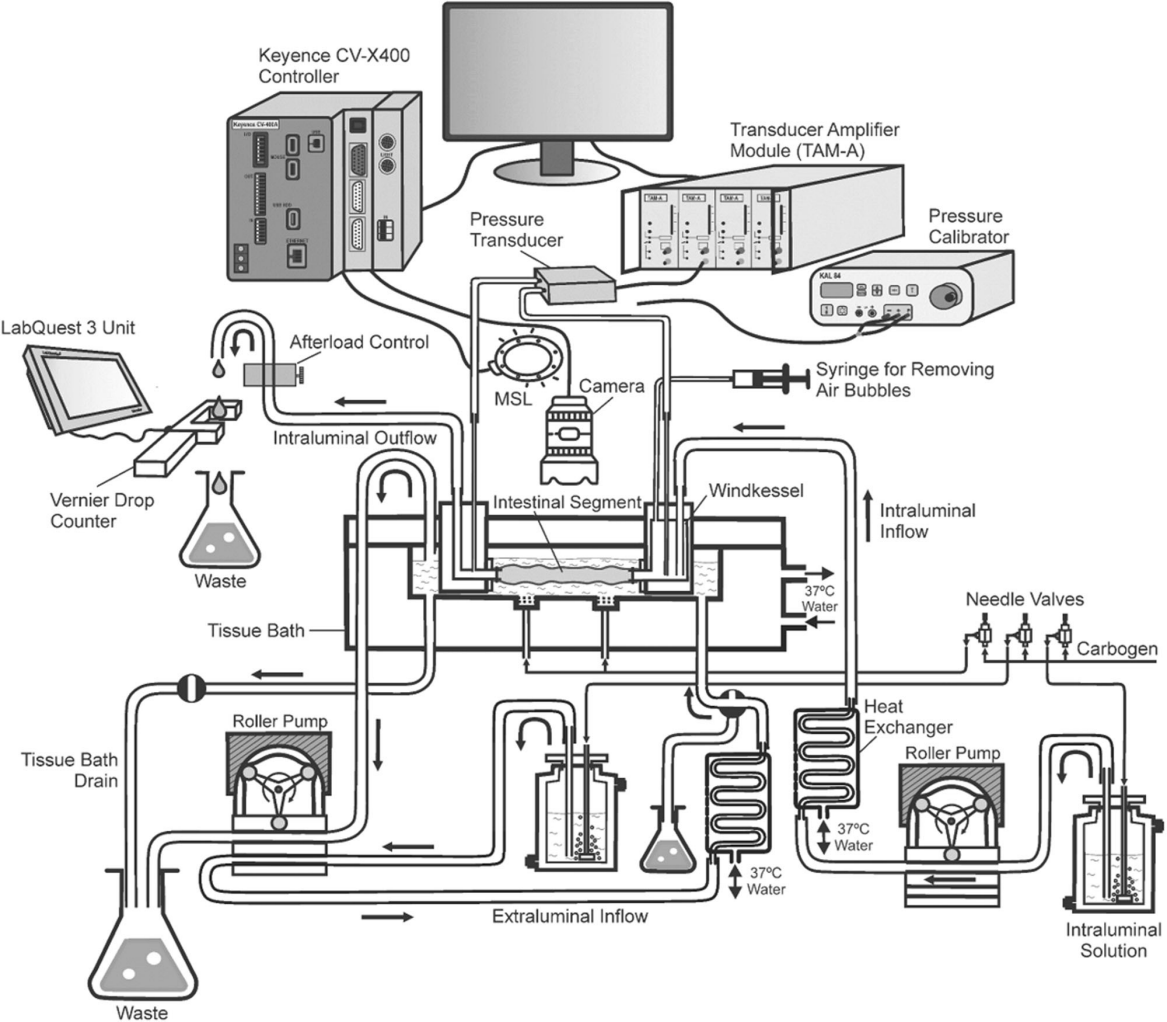
Flow diagram showing the setup for studying peristalsis. The setup involves tissue bath, with units for pressure transducer-amplifier, camera control and droplet discharge sensor-meter. The setup allows for a comprehensive study of intestinal motility capturing movements of the intestinal segment mounted in a perfusion bath using a Keyence camera, multi-spectrum LumiTrax light (MSL) and CV-X400 series controller system. Changes in intraluminal pressure were measured using a pressure transducer and transducer amplifier module (TAM-A) connected to the ports on the tissue bath. A droplet discharge occurring at the aboral end was detected using the infrared-LED sensor drop counter (Vernier Instruments, USA) and metered using LabQuest Mini Interface. Together this setup allows for a comprehensive study of intestinal motility and enhances the understanding of complex biological processes responsible for droplet discharge. The figure was created using Corel Draw, version 21.3.0.755 (2019).

Intestinal peristalsis and DD were studied using jejunal segments from 9–12 week old Swiss Albino male mice. The GI electrophysiology of mice and humans is largely similar and therefore justified their use to study GI motility^18^. GI motility relies on intricate interactions between extrinsic and intrinsic neural networks, including the pivotal role of ICC and smooth muscle cells^39^. Intrinsic neuronal plexuses provide autonomous control over GI function, allowing the intestine to operate independently from extrinsic neuronal inputs^40,41^. This supported the use of isolated intestinal segments in the tissue bath in the present study. All experiments were approved by the University of Florida Institutional Animal Care and Use Committee (IACUC#: 202300000119). Mice were humanely sacrificed by CO_2_ narcosis followed by cervical dislocation (per American Veterinary Medical Association’s Guidelines for the Euthanasia of Animals). Thereafter, a ~4.5 cm jejunal segment located 12 cm proximal to the cecum was identified, dissected and then mounted in a Mayflower tissue bath (Type 813/6), which is a horizontal water-jacketed chamber (Hugo Sachs Elektronik, Harvard Apparatus, USA), held at 37 °C (Fig. 3a–e). Precise length of the intestinal segments was measured after mounting using a Vernier Calliper, and this information was used by the computer program to accurately calculate the edge width and quantify longitudinal movements. Intraluminal perfusion was achieved using a multi-channel roller pump calibrated to deliver Ringer’s solution at a steady state rate of ~0.065 ml/min (Figs. 3a and 4). Ringer was maintained at 37 °C in a water-jacketed glass buffer reservoir (73-3440; Hugo Sachs Elektronik/Harvard Apparatus, Germany) by passing the solution through a heat exchanger^42^. The perfusate was passed through a Windkessel (Figs. 3b and 4) to dissipate any pulsatility coming from the roller pumps. A separate multi-channel roller pump was used to superfuse the intestinal segment at a rate of 0.2 ml/min also at 37 °C. The Ringer solution for both perfusion and superfusion was continuously bubbled with 95% O_2_ and 5% CO_2_ (carbogen) and maintained at pH 7.4 (Fig. 4). Using this setup, the intestinal segments studied stayed viable for ~ 45 min. During this period, the ILP and volume changes were maintained grossly at a steady level. As the tissue deteriorated in the chamber, there were gross reduction in ILP and increase in volume with time. Therefore, most of the studies were limited to the first 30 min. In this setup, the lumen was filled using a peristaltic pump and an afterload was applied to generate an ILP ranging from 0 to 5 cmH_2_O column by increasing the height of the luminal efflux tubing (Figs. 3d and 4), as performed in previous studies^43–45^. Each perfusion pressure was maintained for 10 min and the ILP of 1.5 cmH_2_O was found to have the maximum amplitude pressure waves maintained for the longest period of time. The afterload help exert the mechanical stretch reflex via the enteric nervous system (ENS) that resides within the wall of the GI tract, including intrinsic primary afferent neurons (IPANs), interneurons, and motor neurons to achieve smooth muscle contraction^45,46^. IPANs are sensory neurons that detect the mechanical, hormonal, or chemical changes in the gut and the interneurons transmit the information to the enteric neural network, while the motor neurons provide the desired muscular activity^41^. This triggers mechanically a cascade of alterations in the CM and LM frequency, and contraction patterns, ultimately resulting in DD at the aboral end with different duration.

### Intraluminal pressure measurements

**Fig. 5:**
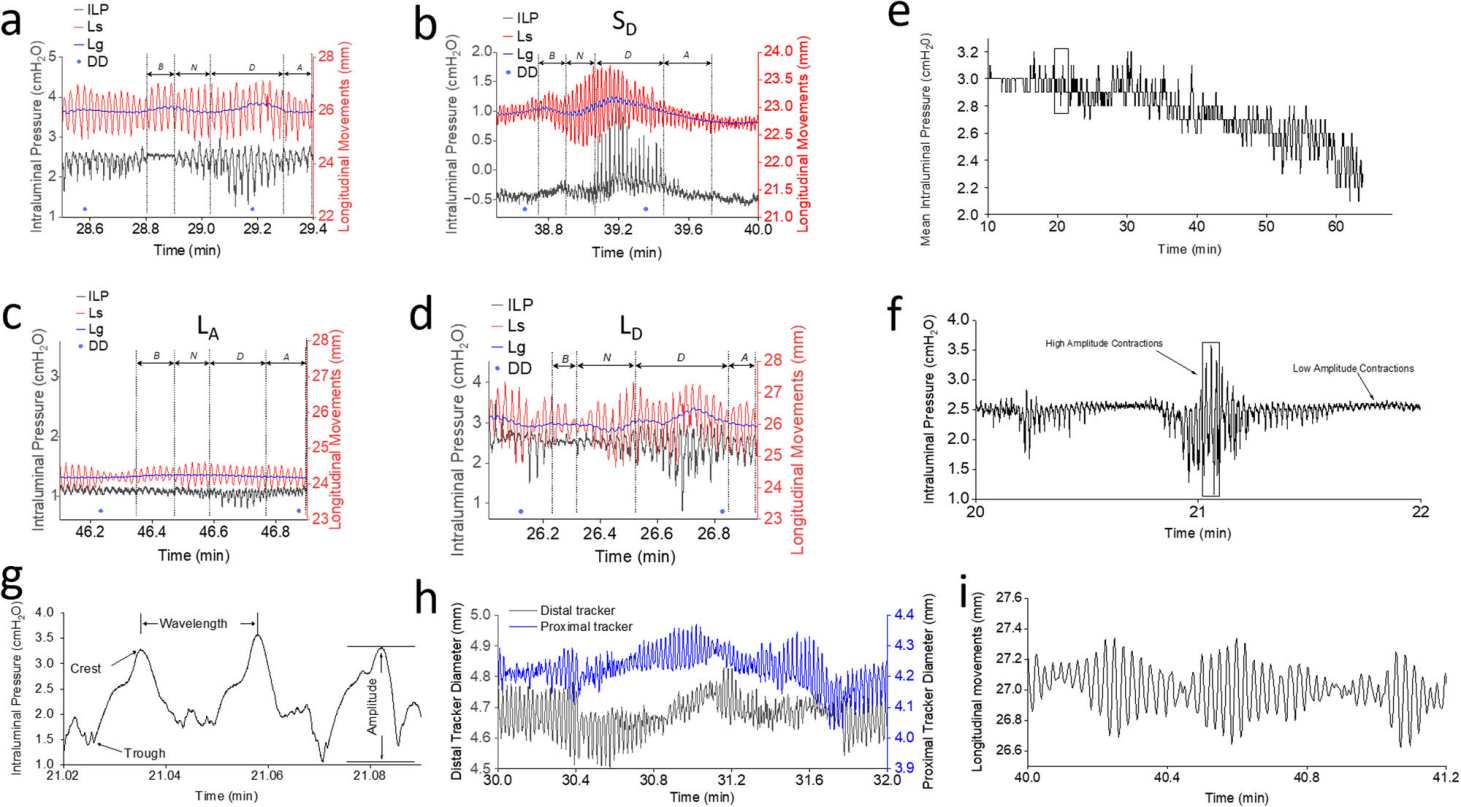
Representative tracings for intraluminal pressure, longitudinal and circular muscle contraction, and droplet discharge. **a** The intraluminal pressure (ILP) tracings showing separation into four phases based on *Ps*, *Ls* and/or *Lg*. *Lg* trace (blue) is obtained by selecting the mean of each *Ls*. “Before-phase” (*B*_Phase_) started with low *Lg* and *Ls*, and both progressively increased by the end of the phase. During *B*_Phase_, low amplitude pressure waves were observed, and this phase was followed by a phase with increased *Ps*, called the “near-phase” or *N*_Phase_. The *N*_Phase_ started with a rapid increase in *Ps*, and during this period *Ls* progressively increased and reached a peak or plateau marking the end of the *N*_Phase_. The *N*_Phase_ displayed slightly higher *Ps* when compared to *B*_Phase_. The *N*_Phase_ preceded the high amplitude *Ps* in “during-phase” (*D*_Phase_). Both, *Ls* and *Lg* progressively increased and reached a peak from the beginning of the *N*_Phase_. The *D*_Phase_ is the region of the pressure tracing where the highest amplitude contraction waves were observed and was significantly higher when compared to *B*_Phase_ and *N*_Phase_. The *D*_Phase_ had the longest duration and was followed by “after-phase” (*A*_Phase_). At the end of the *D*_Phase_, *Ps*, *Pg* and *Lg* returned to the base levels and marked the beginning for the *A*_Phase_. Duration and amplitude (*Ps*) for *A*_phase_ was significantly lower when compared to *D*_Phase_ but was comparable to that of *B*_Phase_. **b** Representative tracings for S_D_. The figure shows a significant increase in longitudinal movements (*Lg* and *Ls*) from *B*_Phase_ to *D*_phase_, during which *Ps* showed a significant increase. DD occurred in *D*_Phase_ (blue dot). In *A*_Phase_, *Lg* and *Ls* decreased together with a decrease in *Ps* and *Pg*. **c** Representative tracings for L_A._ The amplitude of *Ps, Pg, Ls* and *Lg* were relatively smaller when compared to S_D_ and L_D_. *Lg* and *Ls* increased from *B*_Phase_ to *D*_Phase_ and decreased from *D*_Phase_ to *A*_Phase_. DD occurred in *A*_Phase_ (blue dots). **d** Representative tracings for L_D_. An increase in Lg was observed from *N*_Phase_ to *D*_Phase_ but was significantly lower than S_D_ drops. Duration for L_A_ and L_D_ were significantly higher when compared to S_D_. The decrease in *Lg* during the *A*_phase_ in L_D_ was significantly lower than that observed in S_D_ drops. DD occurred in *D*_Phase_. At the end of *A*_Phase_ *Lg* decreased to the levels observed in *B*_phase_. DD occurred in *D*_Phase_ (blue dot). Decrease in *Lg* in *A*_phase_ was observed in all DD groups. **e** Representative pressure trace showing trend over time. ILP maintained steady levels for up to 40 min and thereafter, ILP decreased significantly. Therefore, all subsequent studies were performed within the first 30 min. **f** Zoomed view from (**e**) (20–22 min) showing low and high amplitude pressure waves (*Ps*) with gross pressure changes (*Pg*). Pressure tracings show high amplitude waves flanked by small rise and fall in pressure strength. **g** Zoomed view from (**f**) (21.02–21.1 min) showing crest, trough, amplitude, and wavelength. **h** Representative traces showing the diameter changes in proximal (*EW*_1_) and distal (*EW*_4_) tracker with time. Lower values represent decrease in diameter or circular muscle contraction. **i** Representative trace showing low and high amplitude oscillations (*Ls*) along the gross longitudinal muscle contraction (*Lg*). The *Lg* tracings recorded using PatternTrax are read from left to right, thereby increased values suggest contraction of longitudinal muscles at the proximal end while decreased values suggest contraction at the distal end.

ILP plays an important role in the movement of luminal content through the intestine, and was recorded in the past using high resolution manometry and pressure transducers connected to the oral and aboral ends. In the current study, the oral and aboral ends of the tissues were connected to two individual pressure ports at the top of the perfusion bath by using Tygon tubing with an inner diameter of 0.79 mm attached to a differential pressure transducer MPX (type 399/2) that uses a monolithic silicon piezoresistor (Hugo Sachs Elektronik/Harvard Apparatus, Germany). The pressure was calibrated after adjusting the water column using the afterload control (Fig. 4). This setup therefore accurately delivers pressure changes occurring in the intestinal segment (PBTO)^42^. Such a setup does not impede or alter the natural movement of the intestine. The pressure signal from the transducers were amplified using a universal DC bridge amplifier (Transducer Amplifier module (TAM-A) type 705/1 (Hugo Sachs Elektronik/Harvard Apparatus, Germany; Figs. 3b and 4). Four such TAM-A modules were housed within an HSE Plugsys measuring system. The Plugsys apparatus captured signals originating from the isolated intestinal segments mounted in PBTOs, and amplified and continuously recorded every 2 msec, using a Data Acquisition Hardware and Basic data acquisition software (HSE-BDAS; Hugo Sachs Elektronik/Harvard Apparatus, Germany) running on a Windows 10 operating system. A low-pressure afterload of 1.5 cmH_2_O was applied using a raised water column to simulate intestinal distention during the passage of the food (Figs. 3e and 4). The pressure waves acquired by the BDAS software were too enormous to manually quantify the number of high amplitude waves and their duration. Therefore, a proprietary program, peristalsis.exe^47^ was used to accurately quantify changes in pressure, frequency, amplitude, strength, and duration of contractions. These pressure parameters were later correlated with peristaltic events such as changes in outer diameter, longitudinal movements of the intestine and DD at the aboral end. Predetermined thresholds were set in the program to differentiate low and high amplitude pressure changes, facilitating analysis of large data sets. Our plan going forward was to use this experimental setup to assess segmental differences in ILP, and CM and LM contractions and their corresponding frequency (Fig. 5a–i), with and without various pharmacological interventions intended to mimic GI motility dysfunction in human disease states.

Graphical data obtained using BDAS displayed both low and high amplitude pressure changes (Fig. 5e–g). The frequency of high amplitude contractions in jejunal segments were determined using “Peristalsis.exe”^47^ while low amplitude contractions were assessed manually. The frequencies of both low and high amplitude contractions were similar and not statistically different within each corresponding intestinal segment (0.69 ± 0.01 Hz vs. 0.69 ± 0.01 Hz; *p* > 0.05, *n* = 192). However, there was a significant difference in frequency between the jejunal and ileal segments (0.76 ± 0.01 Hz vs. 0.55 ± 0.01 Hz; *p* < 0.001, *n* = 22). This aligns with prior research indicating that the frequency progressively decreases in the distal regions of the GI tract compared to the jejunum^30,48^. Given the distinct frequency differences in the jejunum and ileum, indicating the involvement of separate pacemaker groups, these segments were individually analyzed to uncover variations in muscular activities responsible for fluid discharge. The present study specifically concentrates on the jejunal gut segment.

ILP recordings between tissues were compared between experiments using “gross pressure” (*Pg*) and “pressure strength” (*Ps*). In the detailed pressure recording window of BDAS, we observed multiple high-amplitude pressure waves occurring at irregular intervals (Fig. 5e–g). However, it was unclear how these waves affected *Pg*. *Ps* of these waves was calculated as the algebraic sum of individual pressure waves amplitudes (measured from crest to trough) divided by the number of waves. While *Pg* in the intestinal segments studied were calculated from the measured ILP recordings (Fig. 5e), and mathematically represented as *Pg* = the sum of pressure recordings taken over the study period divided by the number of recordings taken over the study period. Changes in *Pg* between phases measured the magnitude of pressure changes without considering the oscillations associated with ILP waves.

### Analysis of edge width, volume, longitudinal movement and contraction type

Spatiotemporal mapping has been the most widely used technique to track CM and LM contractions. In this study, to monitor and quantify two-dimensional edge movements for CM and LM contractions in intestinal segments in real-time, we employed an innovative approach utilizing a CV-X400 series vision system. This setup comprised an ultra-high-speed camera (LumiTrax™), multi-spectrum lighting with eight color LEDs, and a dedicated control circuit (Keyence, USA) (Figs. 3a, b and 4). LumiTrax™ employs a novel imaging technique in which the lighting direction and color were automatically synchronized with the camera through machine learning to collect data and quantify real-time changes in both LM (length) and CM contraction (diameter) amplitudes. Multiple images with 24 image enhancement filters and lighting from various directions are taken and analyzed to detect patterns, such as blood vessels or mesentery, on the intestinal surface to optimize real-time visualization and tracking during intestinal movements, even when their orientation changes (Fig. 3c). Build-in “Auto-Teach Inspection Tools” allows the camera to “learn” and identify variations and differences in the pattern that may occur with intestinal contraction in real-time, ensuring stable tracking to obtain quantifiable data acquisition of diameter and longitudinal movements. This capability enables real-time tracking with less noise during data capture. The camera operates with preset configurations, reducing setup time, thus enhancing tissue viability. Both longitudinal and circular movements are captured every 50 msec.

**Fig. 6:**
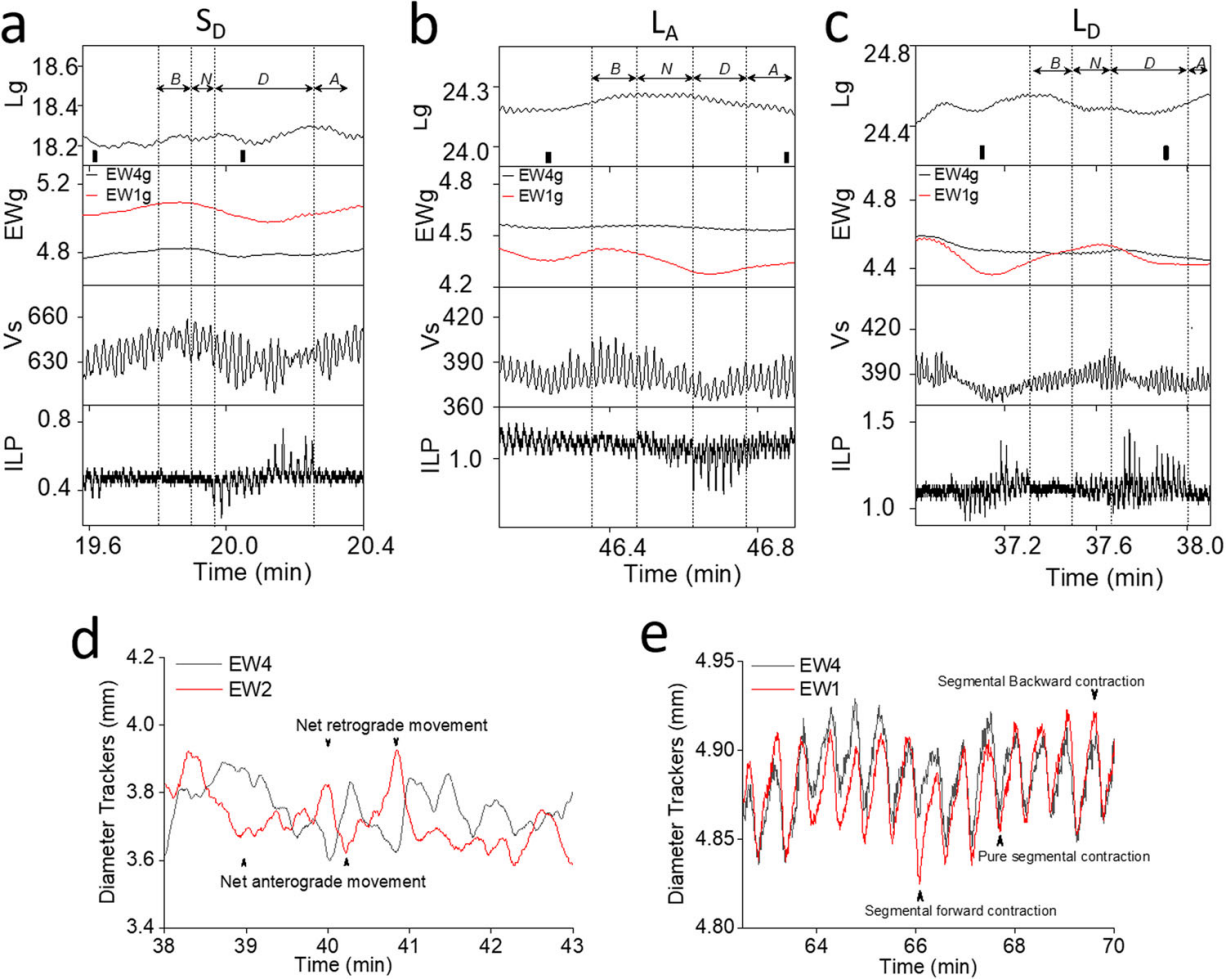
Panel graphs showing traces of factors responsible for droplet discharge duration and segmentation: **a** S_D_ discharge: in *B*_Phase_ to *N*_Phase_, longitudinal muscles showed maximum values, *EW*_1_ and *EW*_4_ begins to contract with maximum reduction occurring in the *D*_Phase_. Longitudinal muscle contraction at proximal end occurred before circular muscle began to contract. *EW*_1_*g* showed greater contraction when compared to *EW*_4_*g*. Correspondingly, maximum increase in volume was seen in *B*_Phase_ and thereafter, the volume began to decrease with maximum reduction in the middle of *D*_Phase_. *Lg*, *EWg* and *Vg* showed reduction in the *D*_Phase_, where *Ps* was increased. *Lg* increased from the middle of *D*_Phase_ and reached a maximum toward the early part of *A*_Phase_ while, *EWg* and *Vg* continued to increase through the *A*_Phase_. *Ps* decreased during the *A*_Phase_. **b** *L*_*A*_ discharge: longitudinal muscles showed an increase from *B*_Phase_ to *D*_Phase_ and thereafter decreased from the beginning of *D*_Phase_ and continued into the *A*_Phase_. *EW*_1_ started to contract in *B*_Phase_, *N*_Phase_ and early *D*_Phase_, while longitudinal muscle remained in the contracted state at the proximal end. *EW*_1_*g* showed a marginal increase in *D*_Phase_ and *A*_Phase_. Unlike *EW*_1_*g*, *EW*_4_*g* contractions were minimal. Volume changes are minimal compared to S_D_. An increase in volume was observed from *N*_Phase_ to *D*_Phase_, followed by a decrease in *A*_Phase_. **c** L_D_ discharge: longitudinal muscle contraction showed a decrease in *B*_Phase_ to *N*_Phase_, unlike S_D_ and L_A_. *EW*_1_*g* values increased from *B*_Phase_ to *D*_Phase_, suggesting continued filling through *B*_Phase_ and *N*_Phase_, and began to empty only in the *D*_Phase_. Unlike S_D_ and L_A_, in L_D_, the lumen continued to fill through *B*_Phase_ and *N*_Phase_. *EW*_4_*g* continued to decrease from *B*_Phase_ to *A*_Phase_ with maximum decrease occurring in *B*_Phase_ to *N*_Phase_. Volume decreased in *D*_Phase_ and did not return to base levels suggesting incomplete emptying. **d** Diameter changes between *EW*_1_ and *EW*_4_ depicting anterograde and retrograde contraction analyzed using “GutCode”. *EW*_1_ contraction with *EW*_4_ relaxation causes anterograde movement (upward arrow), and *EW*_1_ contraction with *EW*_4_ relaxation causes retrograde contraction (downward arrow). **e** Diameter changes between *EW*_1_ and *EW*_4_ depicting segmental contractions analysed by “GutCode”. Simultaneous contraction or relaxation of *EW*_1_ and *EW*_4_ causes pure segmental contraction. A small increase in diameter of *EW*_4_ compared to *EW*_1_ causes segmental forward, and a small decrease in diameter of *EW*_4_ compared to *EW*_1_ causes segmental backward contractions.

Longitudinal movements are tracked and recorded from the aboral end. The gross longitudinal movements (*Lg*) tracked using “PatternTrax” helped determine whether LM contractions were oriented toward the oral or aboral end of the intestine. These movements were recorded as a gross shift along the *Y*-axis (*Lg*) over time (Figs. 5i and 6a–c) and are calculated by subtracting the mean longitudinal position of each phase from the previous phase. Increased *Lg* values indicate LM contractions toward the oral end, while decreased values suggest gross LM movement toward the aboral end. Due to slight differences in the length of the intestinal segment studied and the position of the PatternTrax on the intestine, the baseline *Lg* levels were never exactly the same. Therefore, we normalized the values by subtracting the smallest value from all longitudinal recordings to obtain the absolute distance moved, facilitating comparisons between experiments or different phases of DD. These values reflect the strength of the LM contractions. By comprehensively studying the coordinated roles of LM movements, CM movements, and their influence on ILP changes, we can bridge the existing knowledge gap and gain a deeper understanding of how these factors collectively contribute to the propulsion of luminal contents. LM tracings exhibited both low and high amplitude oscillations with amplitude representing the strength of LM contraction, denoted as *Ls*, calculated as the algebraic sum of the amplitudes of individual longitudinal waves divided by the number of waves. To understand the role of *Ls* in determining the duration of DD and how it interacts with *Lg*, *Ls* was compared across different phases and DD groups (Fig. 5b–d).

For diameter tracking, we selected opposite edges of the intestine at four regions along its length as edge width (*EW*) trackers using the CV-X400 series machine vision system. These EW trackers measured changes in the distance between opposite edges during contraction generating a graphical representation of diameter changes. All four pairs of EW trackers were used continuously along the sides of intestinal segment, with adjacent trackers spaced apart by approximately the length of one edge tracker. Contraction was indicated by a decrease in EW (diameter), while relaxation was indicated by an increase. The diameter measurement from the four EW trackers changed with changes in fluid volume in the intestine. The analysis of the gross movement of EW trackers (*EWg*) depicts the overall diameter change, which is calculated by subtracting the mean diameter in one phase from that of the previous phase. The amplitude of EW contractions at any given time represents the strength of EW contractions (*EWs*). To ensure that the changes recorded by “GutCode” accurately represented the actual variations in diameter in each of the EW trackers, the magnitude of contraction in each phase was manually measured. The data obtained through “GutCode”^49^ aligned well with the gross movement of the proximal edge width tracker (*EW*_1_*g*). The gross movement of edge width tracker contraction waves was calculated by dividing the sum of all the crests by the duration of the time period studied, and this measured the magnitude of the contraction. *EW*_1_*g* traces showed low and high amplitude oscillations, and the amplitude of the oscillation was considered as the strength of the contractions, and is denoted as *EW*_1_*s*. To determine the role of *EW*_1_*s* in the duration of DD, *EW*_*1*_*s* was compared between different DD groups.

The propagation of waves along the intestinal wall was captured using four pairs of *EW* trackers. The propagation of contraction waves along the intestinal wall and the development of anterograde, retrograde, and segmental contractions play a crucial role in intestinal motility and the dynamics of DD (Fig. 6a–e). The proximal tracker contraction with a distal tracker relaxation resulted in anterograde propulsion while a distal tracker contraction with a proximal tracker relaxation generated retrograde propulsion. When both proximal and distal trackers contracted or relaxed simultaneously, the contraction waves neither proceeded orally nor aborally, triggering segmental contraction (Fig. 6d, e).

The changes in EW trackers were used to calculate real time changes in intestinal volume (*V*) using the formula *V* =  $\pi \mathop{\sum }\nolimits_{i=1}^{4}{hi}\frac{{d}_{i}^{2}}{4}$, where *d*1, *d*2, *d*3, and *d*4 represent the diameters recorded by corresponding EW trackers, and *h*1, *h*2, *h*3, and *h*4 represent the length of the intestine measured by the trackers, including half the gap between trackers on each side of the gut segment. The formula for volume calculation was integrated into the proprietary program “GutCode”^49^ which automated and expedited the computation of diameter and volume changes from EW tracings. However, it’s important to note that this volume calculation represents volume changes and could be influenced by the longitudinal and circular muscle contractions, as it was not possible to separate the contributions of circular and longitudinal muscle contractions in our method. The volume calculation helps us understand how luminal filling or emptying influenced DD and ILP changes, or CM and LM movements. Volume tracings showed low and high amplitude contraction waves, and the amplitude of these waves was described as volume strength (*Vs*) and the gross volume movement was described as *Vg*.

### Quantification of anterograde, retrograde and segmental contractions

We utilized “GutCode”^49^ for the analysis of diameter data obtained from four EW trackers positioned along the oral-to-aboral axis of intestinal segments. Contractions were categorized into anterograde, retrograde, and segmental based on relative movements of adjacent trackers. Anterograde was noted when the proximal tracker detected contraction followed by relaxation detected by the distal tracker. Retrograde was identified when the proximal tracker noted relaxation followed by contraction detected by the distal tracker.

Segmental contractions were recognized when both proximal and distal trackers detected contractions simultaneously. To quantify contraction strength, we divided the sum of the amplitudes by time, assigning positive values to anterograde, negative values to retrograde, and segmental values based on their overall inclination toward anterograde or retrograde with a positive or a negative sign.

Since “GutCode”^49^ processed data obtained at a 50 msec intervals, and contraction-relaxation cycle occur at 0.7 Hz, where each wave takes ~1.4 s to complete, only minor shifts were analyzed in anterograde, retrograde, or segmental contractions between *EW* trackers for each data point. In addition, the slow waves generating propulsive movements can instantaneously change direction and speed of propagation^50^. Therefore, to determine the overall contraction direction, we introduced “net amplitude”, accounting for small amplitude shifts among all four trackers. A “net amplitude” > 0 indicated anterograde, while <0 indicated retrograde contractions. “Net amplitude” allowed for comparisons between different discharge groups and contraction phases. These recordings provided insights into various contractions, tabulating their frequency, strength, duration, amplitude, retrograde, segmental, and anterograde characteristics, and their impact on intestinal DD. This tabulation facilitated easy comparisons between studied tissue segments (Figs. 1a–d, 5a–i, 6a–e and Tables 1 and 2).

### Experimental analysis of droplet discharge

The outflow from the luminal perfusate that constituted a DD was directed to pass through a 1.3 × 3.7 cm opening of Vernier Drop Counter, equipped with an infrared (890 nm) LED emitter at one end, and a detector at the opposite end. When a drop of perfusate obstructed the infrared beam between the emitter and detector, a digital signal was transmitted to the LabQuest®3 system. These drops were then converted into microliters (μl) using a calibration chart within the LabQuest®3 program (Figs. 3d and 4). This method allowed for precise and automated measurement of the time and volume of each DD event occurring at the aboral end in the studied intestinal segments. Thus, DD duration was assessed as the interval between two DD.

Rate of fluid flow through the tubing and/or intestinal segment was represented as “Flow rate”. The flow rate was calculated by dividing the volume of a drop obtained at the aboral end by the time elapsed between DD events, and was represented as ml/min. The flow rate exhibited slight variations between experiments due to minor differences in flow arising from the peristaltic pump and tubing employed. The average flow rate was 0.065 ml/min. The flow rate through the intestine was therefore determined by running the perfusate through a Tygon tubing in lieu of the intestinal segment, both before and after the experiment, and the mean was represented as the average flow rate. The flow through the intestinal segment or the Tygon tubing was achieved using a peristaltic pump (Figs. 3a and 4). Accurate determination of DD event duration is crucial for precise muscular event evaluation. To account for slight variations in flow duration, we normalized the experimental duration (D) for each DD event while the intestine was positioned in the Mayflower bath by dividing D by the standard duration (Ds) when the perfusate passed through a Tygon tubing. Consequently, we utilized the normalization method D/Ds to mitigate potential variations in the flow rate introduced by the pump and tubing during the experiment. When the D/Ds ratio approached one, it indicated minimal variations.

The total inflow and outflow volume over a 15-min period was calculated, and their difference determined the secretory or absorptive nature of the studied intestinal segment. An increase in outflow volume indicated secretion, whereas a decrease suggested absorption. The variations in D/Ds were compared to other muscular events such as changes in ILP, longitudinal movements, diameter (i.e., *EW* contractions). This analysis provided insights into the mechanisms governing fluid flow and discharge at the aboral end of the gastrointestinal tract.

Since pressure waves were a reflection of CM and /or LM contractions, they were effectively employed as a reference to deduce DD, and assess all muscular activities. When categorizing DD duration, it was observed that DDs could be either short or long in duration. These distinctive durations were closely associated with various muscular parameters and amplitudes of the pressure waves. Therefore, when DDs were sorted by duration, discharges were found to occur either in *D*_Phase_ or *A*_Phase_ (Fig. 5a). Among the 48 DDs studied, the shortest duration DDs primarily occurred in *D*_Phase_, making up 37.5% of total discharges and constituting the S_D_ group (Fig. 5b). Majority of DDs (39.6%) with longer durations occurred in *A*_Phase_, and were represented as the L_A_ group (Fig. 5c). Additionally, 16.7% of DDs occurring in *D*_Phase_ had longer durations, classified as the L_D_ group (Fig. 5d). Three DDs, which occurred in *A*_Phase_ but had shorter durations compared to L_A_, and one discharge occurring in both *D*_Phase_ and *A*_Phase_ with a long duration, were excluded from further analysis. Thus, based on duration and D/Ds, we identified three discharge groups: S_D_, L_A_, and L_D_.

To gain further insights, we divided the pressure contractions during each DD into four distinct phases (Fig. 5a) based on the *Ps*. Pressure levels ranging from 0.03 to 0.2 cmH_2_O were considered low amplitude pressure waves, while pressures exceeding 0.2 cmH_2_O indicated high amplitude contractions. These high amplitude pressure waves were further categorized based on *Ps*, *Ls*, and/or *Lg*. These parameters exhibited significant changes during these high amplitude pressure waves:

- The *B*_Phase_ featured low Ls and Lg, both of which gradually increased throughout this phase. During the *B*_Phase_, low amplitude pressure waves were observed.
- The *N*_Phase_ began with a rapid Ps increase following *B*_Phase_. During this phase, *Ls* progressively increased and reached a peak or plateau which marked the end of the *N*_Phase_. *Lg* did not increase as much as in *B*_Phase_ or in some instances it showed a small decrease. The increase in *Lg* from *B*_Phase_ to *N*_Phase_ and *N*_Phase_ to *D*_Phase_ suggests LM movement and contraction at the oral end. Conversely, the decrease in *Lg* in *A*_Phase_ suggests LM movement and contraction at the aboral end.
- The *D*_Phase_ followed the *N*_Phase_ and was characterized by progressively increasing Ls and Lg, both reaching a peak. This phase exhibited the highest amplitude pressure contractions. *D*_Phase_ showed pressure tracings with the highest amplitude contraction waves and was significantly higher when compared *N*_Phase_ (*p* < 0.001, *n* = 48). *D*_Phase_ had the following characteristics: (1) Increase in *Ps* and reaching a plateau that marks the end of *D*_Phase_; (2) Increase in *Ls* and *Lg* followed by a decrease, which paralleled changes in *Ps*. Thus, at the end of the *D*_Phase_, *Ps*, *Pg* and *Lg* return to baseline levels, which marked the beginning of the *A*_Phase_ (Fig. 5a).
- The *A*_Phase_ succeeded the *D*_Phase_. It was shorter in duration and featured lower amplitude pressure contractions compared to *D*_Phase_, resembling *B*_Phase_ in characteristics. The total duration (20.1 ± 0.1% vs. 45.7 ± 0.8%) and amplitude (0.23 ± 0.02 cmH_2_O vs. 0.66 ± 0.07 cmH_2_O; *p* < 0.001, *n* = 48) for *A*_Phase_ was significantly lower than *D*_Phase_. *A*_Phase_ was comparable to *B*_Phase_ (Fig. 5a).

These phases provided valuable information about the relative movement and contraction of LM and CM, as well as their timing. This enhanced our understanding of the muscular activity that initiates or precedes each contraction.

### Statistics

Statistical analysis was conducted using OriginPro 9.9 (2022), and the different phases were compared within each discharge group. The data were presented as mean ± SEM, and the range of the data sets for DD was shown. Normality of the data sets was evaluated with Shapiro–Wilk test. One-way ANOVA was employed, followed by post hoc Bonferroni test to determine significant differences of data between the four phases. Kruskal–Wallis test was used for overall comparison of the discharge groups, and post hoc Mann–Whitney test was utilized to compare discharge groups between experiments. A significance level (*p*) was set as <0.05.

### Reporting summary

Further information on research design is available in the Nature Portfolio Reporting Summary linked to this article.

### Supplementary information

Peer Review File

Reporting Summary

## Data Availability

All raw data that support this study are available as Supplementary Data and software files in https://zenodo.org/^51^.

## Code availability

The software applications used to extract and calculate the data points are available as Supplementary Data and software files in https://zenodo.org/^47,49^.
